# Updates on the Role of Molecular Alterations and NOTCH Signalling in the Development of Neuroendocrine Neoplasms

**DOI:** 10.3390/jcm8091277

**Published:** 2019-08-22

**Authors:** Claudia von Arx, Monica Capozzi, Elena López-Jiménez, Alessandro Ottaiano, Fabiana Tatangelo, Annabella Di Mauro, Guglielmo Nasti, Maria Lina Tornesello, Salvatore Tafuto

**Affiliations:** 1 Department of Abdominal Oncology, Istituto Nazionale Tumori, IRCCS Fondazione “G. Pascale”, 80131 Naples, Italy; 2 Department of Surgery and Cancer, Imperial College London, London W12 0HS, UK; 3 Cancer Cell Metabolism Group. Centre for Haematology, Immunology and Inflammation Department, Imperial College London, London W12 0HS, UK; 4 SSD Innovative Therapies for Abdominal Metastases—Department of Abdominal Oncology, Istituto Nazionale Tumori, IRCCS—Fondazione “G. Pascale”, 80131 Naples, Italy; 5 Department of Pathology, Istituto Nazionale Tumori, IRCCS—Fondazione “G. Pascale”, 80131 Naples, Italy; 6 Unit of Molecular Biology and Viral Oncology, Department of Research, Istituto Nazionale Tumori IRCCS Fondazione Pascale, 80131 Naples, Italy

**Keywords:** neuroendocrine neoplasms, NOTCH, cancer-driven genes, mutational mechanism, germline mutations, small cell lung carcinoma, pancreatic NET, small bowel NET, medullary thyroid carcinoma, malignant castration-resistant prostatic cells

## Abstract

Neuroendocrine neoplasms (NENs) comprise a heterogeneous group of rare malignancies, mainly originating from hormone-secreting cells, which are widespread in human tissues. The identification of mutations in ATRX/DAXX genes in sporadic NENs, as well as the high burden of mutations scattered throughout the multiple endocrine neoplasia type 1 (MEN-1) gene in both sporadic and inherited syndromes, provided new insights into the molecular biology of tumour development. Other molecular mechanisms, such as the NOTCH signalling pathway, have shown to play an important role in the pathogenesis of NENs. NOTCH receptors are expressed on neuroendocrine cells and generally act as tumour suppressor proteins, but in some contexts can function as oncogenes. The biological heterogeneity of NENs suggests that to fully understand the role and the potential therapeutic implications of gene mutations and NOTCH signalling in NENs, a comprehensive analysis of genetic alterations, NOTCH expression patterns and their potential role across all NEN subtypes is required.

## 1. Introduction

Neuroendocrine cells are sensor cells, which play an important role in the connection between the nervous system and endocrine organs. In response to neurogenic stimulation, neuroendocrine cells secrete several molecules, including peptide hormones, which produce slow and long-lasting effects. Neuroendocrine cells are widely scattered throughout the human body. They are present in the gastro-entero-pancreatic tract, uro-genital apparatus, lung, breast and skin, as well as in the central and peripheral nervous system. These cells are able to dedifferentiate and transdifferentiate under physiological conditions in response to intracellular metabolic pathways and microenvironmental stress conditions [1].

NOTCH signaling is a highly conserved cell-signaling pathway that is implicated in different stages of development through the regulation of cell proliferation, differentiation and cell death.

In the neuroendocrine system, NOTCH signaling drives the maturation process of multi-potent cells to become functionally competent cells during the early stage of embryonic neuroendocrine development [2]. For instance, NOTCH signaling regulates the ductal and endocrine differentiation of pancreatic cells during the development of the pancreas [3].

Neuroendocrine neoplasms (NENs) are originated from the neoplastic transformation of neuroendocrine cells at various anatomic locations, with the gastrointestinal tract, the endocrine pancreas and the respiratory tract being the most involved sites. [4].

Little is known about the mechanisms of oncogenic transformation and metastatic dissemination of neuroendocrine cells, but it is known that despite some common molecular characteristics, NENs originating in different organs have distinct signatures and display significant biological heterogeneity.

In this heterogeneous neoplastic setting, the NOTCH pathway has shown to have a role by triggering both tumour suppressor and oncogenic functions in some neuroendocrine cell lines and in different subtypes of NENs [5,6,7,8,9,10].

The availability of treatments with a modulatory activity on NOTCH-dependent pathways, and the possibility to use the molecular alterations as diagnostic and prognostic markers, has highlighted the need of a deeper knowledge on the NOTCH pathway role and the different molecular signatures in NENs.

This review will summarize the current knowledge on the molecular heterogeneity of NENs and the complex function of NOTCH signalling in different types of NENs, as well as the new therapeutic approaches based on NOTCH pathway modulation.

## 2. Neuroendocrine Neoplasms and Molecular Heterogeneity

Neuroendocrine neoplasms are genomically and clinically heterogeneous. This heterogeneity occurs between cancers originating from different organs, within the cancers originating in the same organ, and between primary and metastatic lesions [4,11,12,13]. For instance, small intestine NENs are genomic stable cancers, with a low mutational load compared with NENs originated from different organs, such as the lung and pancreas. Viral-associated Merkel carcinomas have a low mutational burden, in contrast to ultraviolet (UV)-induced Merkel cell carcinomas [14]. The full understanding of the molecular mechanisms and the clinical significance of this heterogeneity could lead to the identification of new hallmarks to target in the neuroendocrine neoplasms’ treatment.

Current advances in genomic analysis techniques have enabled to identify recurrent mutations and chromosomal aberrations at the base of the molecular landscape of NENs [15,16].

Recurrent mutations have been identified in multiple endocrine neoplasia type 1 (*MEN1*) and von Hippel–Lindau (*VHL*) genes, in chromatin remodelling genes, such as *DAXX* and *ATRX*, in mechanistic target of rapamycin (mTOR) pathway genes, especially in phosphatase and tensin homolog (*PTEN*), tuberous sclerosis complex 2 (*TSC2*), and phosphatidylinositol-4,5-bisphosphate 3-kinase catalytic subunit alpha (*PIK3CA*), in checkpoint kinase 2 (*CHEK2*) tumour suppressor gene, in telomerase maintenance genes, in the cell cycle regulator cyclin dependent kinase inhibitor 1B (*CDKN1B*) and in the DNA repair gene mutY DNA glycosylase (*MUTYH*) [15,16]. These mutations can occur in genetic syndromes, such as multiple endocrine neoplasia type 1 (MEN1), tuberous sclerosis complex (TSC1/2), neurofibromatosis type 1 (NF1), and von Hippel–Lindau (VHL) syndrome, or in sporadic NENs, and can be germline or somatic mutations [15,16,17]. Genetic syndromes account for 15–20% of NENs, while the remaining 80–85% are sporadic.

Interestingly, as confirmation of the high heterogeneity of NENs, whole exome sequencing analysis performed in different studies has identified only 21 genes commonly altered between the small intestinal NENs samples analysed from different patients [11,18]. Furthermore, comparing the results of these studies on small bowel NENs with the one on pancreatic NENs, a concordance of only 17 genes with somatic mutations was found [11,15,18].

In addition, some mutations, namely mutations in *MEN1* and *DAXX/ATRX* genes, are associated with a better prognosis, and they seem to occur very rarely in poorly differentiated neuroendocrine carcinomas (NECs) [19]. On the other hand, mutation in *TP53*, *RB1*, *PTEN* and *PIK3CA* are more frequent in poorly differentiated NECs [19,20].

In the following paragraph, we summarise the current knowledge and the clinical significance of the most common genetic alterations in NENs, classifying them according to their hereditary or sporadic condition.

## 3. Common Genetic Alterations and Molecular Pathways in the Development of Neuroendocrine Neoplasms

### 3.1. Heritable Genetic Traits in Neuroendocrine Neoplasms

NENs comprise at least ten recognized inherited NEN syndromes, including multiple endocrine neoplasia type 1 and 2 (MEN-1 and MEN-2), von Hippel–Lindau syndrome (VHL) and neurofibromatosis type 1 (NF1) [21].

MEN-1 is a rare autosomal dominant syndrome caused by inactivating mutations in the MEN-1 gene, and mostly associated with the appearance of neoplastic lesions in the pancreas and duodenum, as well as in pituitary and parathyroid glands [22,23]. The majority of germline mutations in the MEN-1 gene cause the truncation or absence of the menin protein in cancer cells. Typically, tumour development is associated with the mutation of both MEN-1 alleles, however, an incomplete inactivation of this gene has been observed in thymic and duodenal NETs [24,25]. The menin protein is usually located in the nucleus, cytoplasm and around telomeres. However, its specific biological role has not yet been described [26].

MEN-2 syndrome is an inherited autosomal dominant disorder comprising MEN-2A (55% of all cases), MEN-2B (5–10%) and familial medullary thyroid carcinoma (FMTC; 35–40%) [27]. The MEN-2A and MEN-2B patients have almost 100% risk of developing MTC and about 50% risk of developing pheochromocytoma and parathyroid adenomas. MEN-2 syndrome is caused by mutations in RET proto-oncogene, encoding a tyrosine kinase receptor. These mutations cause activation of RAS/MAPK (mitogen-activated protein kinases) and PI3K/AKT (phosphatidylinositol 3-kinase/Protein Kinase B) signalling pathways [28] and may occur in two different regions of the RET gene, originating two different types of disorders. In addition, the familial MTC (FMTC) syndrome, which is also caused by RET mutations, is only associated with MTC, but is less aggressive than MEN-2 tumours [29].

MEN-4 is a rare autosomal dominant syndrome predisposed to NETs development, such as parathyroid and pituitary adenomas, associated with the germline mutations in CDKN1B genes encoding the p27kip protein [30]. However, more studies are needed to know the penetrance and biological effect of CDKN1B mutations in these patients.

Von Hippel–Lindau (VHL) syndrome is associated with pheochromocytomas, paragangliomas and pancreatic neoplasia, and is caused by the loss of the VHL tumour suppressor gene, regulating the hypoxia-inducible factor (HIF) and vascular endothelial growth factor (VEGF) pathways [31,32,33]. The VHL protein shuttles between the nucleus and cytoplasm, binding to elongen C, elongen B, Cullin-2 (Cul2), and RING-box protein 1 (Rbx1) and degrading the alpha subunits of HIF in an oxygen-dependent manner [32,34,35]. Lack of degradation of this factor due to the absence of the VHL protein results, for instance, in an uncontrolled production of factors promoting blood vessel formation (e.g., VEGF) and is implicated in tumour development. The germline mutations in the VHL gene are extremely heterogeneous and are spread throughout the coding sequence. They are present in virtually all families with VHL syndrome, although the exact molecular mechanism of development of NETs in VHL has still many unknowns [36].

Neurofibromatosis type 1 (NF1) syndrome is another familiar neuroendocrine tumour (NET) disorder, which is associated with duodenal NETs or pheochromocytomas and is linked to RAS and ERK/MAPK pathways’ deregulations [37]. Genetic alterations of the NF1 gene include missense, nonsense and splice site mutations, as well as insertions/deletions (in/dels) and chromosomal rearrangements [38]. Tuberosclerosis gene TSC1 (9q34) and TSC2 (16p13.3) are regulated by neurofibromin through mTOR activation, linking the three proteins in terms of their potential roles in tumour progression [37].

Loss of function of the NF1 gene causes mTOR activation and tumour development. Disruption of TSC2 in pancreatic beta cells induces beta cell mass expansion in an mTOR-dependent manner [39]. Furthermore, it has recently been demonstrated that patients with pancreatic NET (pNET), and loss of PTEN protein, as well as tuberosclerosis 1 protein, show a significantly shorter survival [40].

Familial pheochromocytoma and paraganglioma syndromes are autosomal-dominant disorders caused mostly by germline mutations in the succinate dehydrogenase subunit (SDH) genes, such as SDHB, SDHC, SDHD, SDHA, and SDHAF2 (succinate dehydrogenase complex assembly factor 2). These are encoding factors required for the assembly of the mitochondrial complex II [32,33,41,42,43,44,45,46,47,48,49,50]. This mitochondrial complex participates in two main cellular processes: the Krebs cycle and the electron-transport chain. The mutations in the key components for the formation of complex II decrease the enzymatic activity of the rest of the complex. The link between the perturbation in complex II and tumorigenesis still has many unknowns. SDH deficiency leads to pseudohypoxic conditions in cancer cells. However, this fact alone is probably not sufficient to induce the tumorigenic process, and thus different possibilities appear to be feasible; for instance, the implication of ROS or the possibility of the inhibition of other α-ketoglutarate-dependent enzymes.

SDH mutations are commonly associated with multiple pheochromocytomas and paragangliomas. However, gastrointestinal stromal tumours, SDH-deficient renal cell carcinoma and pituitary adenomas can also be associated with these mutations [51,52].

### 3.2. Genetic Alterations and Tumour Mutation Burden in NENs

Several chromosomal alterations and gene mutations have been consistently identified in different types of sporadic NENs, although the tumour mutational burden is relatively low compared to other tumour types [21]. In fact, massive parallel sequencing showed that only 24 cancer driver genes are affected by non-synonymous mutations in neuroendocrine neoplasms [53]. Remarkably, cancer driver genes and mutations are unevenly distributed in different tumour types and may contribute to the mechanisms of NEN heterogeneity. The factors encoded by these mutated genes may affect several pathways involved in cell proliferation, metabolism and chromatin modification.

The genetic landscape of gastro-entero-pancreatic neuroendocrine neoplasms (GEP-NENs) confirmed the essential differences of mutational profiles between well-differentiated NETs, including those with a high proliferation index, and NECs [54].

Mutations in TP53 and RB1 genes are pivotal drivers in poorly differentiated NECs of any anatomical origin [16,55,56,57,58]. Mutations in the TP53 gene have been consistently detected in poorly-differentiated GEP-NECs, with a frequency ranging from 20% to 73% of the tested patients [20,55,59,60]. The presence of TP53 mutations in GEP-NECs correlates with poor survival [20], and recently Ali et al. have demonstrated that p53 immunoexpression in colorectal NECs correlates with a poorer response to platinum-based chemotherapy and worse prognosis [61]. These results suggest a potential diagnostic, prognostic and predictive role of p53 immunoexpression in GEP-NECs, one that is currently under investigation in different trials.

The inactivation of RB1 gene product, which occurs mainly by somatic mutations, has been reported in 71% of poorly differentiated pancreatic NECs [54].

KRAS mutations have been identified in gastric, pancreatic and colorectal NECs with frequencies ranging from 8% to 60% [20,54,60,62,63,64,65,66].

On the other hand, BRAF mutations were only found in colorectal NECs with a frequency between 13% and 59% [67], as well as APC affecting some cancer cases [68].

The genetic alterations characterizing poorly differentiated NECs are absent in Grade (G) 3 NETs. This subtype presents typical mutations of G1/G2 NETs. For example, G3 pancreatic NETs showed high frequency of MEN1, DAXX, and ATRX mutations or protein loss (31–44%, 9–25% and 18–36%, respectively). DAXX and ATRX mutations also significantly correlate with the presence of mutations in mTOR regulators and were associated with poor prognosis in the G2 NETs [16]. Therefore, the scientific community is proposing these mutations as possible biomarkers to distinguish G3 pancreatic NETs from NECs [69]. This has a particular clinical relevance, due to the fact that NECs and G3 NETs are detected at an advanced stage.

The molecular similarities between G1/G2 and G3 NETs suggested a new model for GEP-NEN tumorigenesis in which poorly differentiated NECs and well-differentiated NETs, including G3 NETs, were originated from a common-normal neuroendocrine progenitor through different routes [70]. These foresee the alteration of TP53 and RB1 for all poorly-differentiated NECs, and specific alterations for well-differentiated pancreatic NETs and small intestine NETs [71].

Despite the remarkable biological heterogeneity of NETs, the mammalian target of rapamycin (mTOR) molecular pathway has been found to be prominently altered in a vast majority of NETs [72]. mTOR is a kinase-dependent signalling cascade, formed by the mammalian target of rapamycin complex 1 and 2 (mTORC1 and mTORC2), whose main function is related to controlling cell growth. Mutations in NF1, TSC2 or PTEN-encoding for key suppressor genes of this pathway, and altered expression of the mTOR pathway components, are common hallmarks of a great proportion of NETs, wherein these alterations seem to be directly related with tumour development and progression [72].

Recent evidence pointed out a key role of the NOTCH signalling pathway in NEN development, progression and heterogeneity [73]. Loss-of-function mutations in NOTCH family genes, particularly in NOTCH1, have been identified in human and mouse small cell lung cancer (SCLC) and in neuroendocrine pancreatic cells [58,74]. The integrity of NOTCH components is very important for the proper signalling transduction across the pathway. The canonical Notch cascade needs to receive a signal and be able to act in a ligand-receptor manner for communicating the signal inside the cells. This signal translocation promotes several transcriptional changes in the cellular program that allow cells to perform different functions. With an obstacle on this cell signalling cascade, the cells have to deal with unexpected changes in their programs, usually due to different mutations that interfere in the adequate cell–cell communication or in the transcriptional regulation within the cell.

For a better understanding on how these genetic mutations or genomic alterations could lead to the development of neuroendocrine tumours, in the following section, the composition of NOTCH receptors and the main elements involved in the NOTCH signalling transduction will be explained in detail.

## 4. Structure of NOTCH Receptors and the NOTCH Signalling Pathway

The NOTCH receptor family in mammals comprises four transmembrane proteins (NOTCH1–4), which are evolutionarily conserved with a high homology between different species. NOTCH receptors are activated by trans-ligands expressed on neighbouring cells, whereas cis-ligands within the same cell inhibit the NOTCH signalling [75].

**Figure 1:**
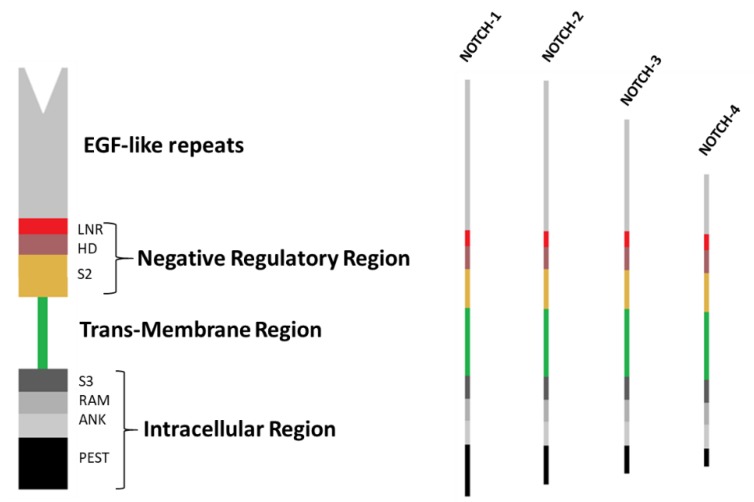
Structure of four human NOTCH receptors: NOTCH receptors are composed of an extracellular region of repetitive epidermal growth factor (EGF)-like sequences (29–36 repeats), a negative regulatory region, a single transmembrane portion and an intra-cytoplasmic tail involved in signal transduction. S2 and S3 are, respectively, the metalloprotease and *γ*-secretase sites of cleavage. EGF: epidermal growth factor; LNR: cysteine-rich LIN12-NOTCH repeats; HD: heterodimerization domain; RAM: recombining binding protein suppressor of hairless (RBPJ) associate module; ANK: ankyrin repeats; PEST: region rich in proline (P), glutamine (E), serine (S) and threonine (T) residues.

The four NOTCH receptor isoforms in mammals are characterized by an extracellular region of repetitive epidermal growth factor (EGF)-like sequences, which are involved in the interaction with delta-like ligands (DLL1, DLL3, DLL4) and jagged proteins (JAG1, JAG2), by a negative regulatory region (NRR) that prevents Notch activation in the absence of the correct signal, by a single transmembrane portion and by an intra-cytoplasmic tail involved in the signal transduction (Figure 1). The number of EGF-like repeats varies between the four NOTCH receptors, being 36 for NOTCH-1 and 2 receptors, 34 for NOTCH-3 and 29 for NOTCH-4. The NRR consists of three cysteine-rich LIN12-NOTCH repeats (LNR) and a heterodimerization domain (HD). The intracellular domain is composed of a recombining binding protein suppressor of hairless (RBPj) associate module, ankirin repeats (ANK) and a C-terminal region rich in proline (P), glutamine (E), serine (S) and threonine (T) residues (PEST). S2 and S3 regions are, respectively, the metalloprotease and *γ*-secretase sites of cleavage. The expression of these receptors is in a cell- and tissue-type specific manner.

**Figure 2:**
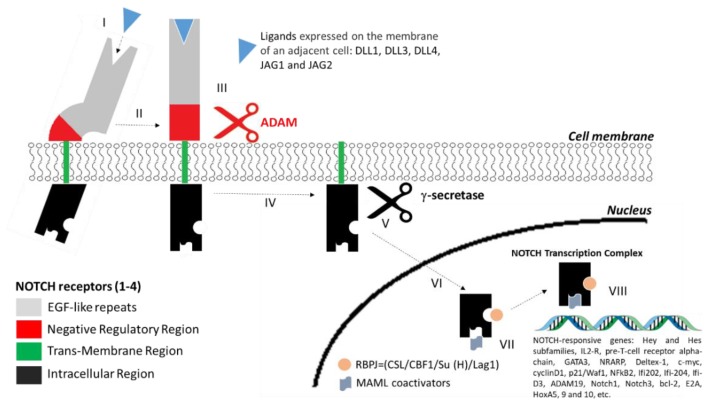
Schematic representation of NOTCH signalling pathway: Sequential steps in the NOTCH signalling pathway are shown as follows: I: NOTCH receptor binding to specific ligands; II: conformational change of the receptor; III: ADAMs-mediated cleavage; IV: recognition of the intracellular region by *γ*-secretase; V: *γ*-secretase mediated cleavage; VI: nuclear translocation; VII: binding to RBPJ and MAML; VIII: transcriptional activation DLL: delta-like ligands; JAG: jagged protein; ADAM: a disintegrin and metalloprotease; RBPJ: recombining binding protein suppressor of hairless; MAML: Mastermind-like co-activators.

The interaction between NOTCH receptors and their ligands initiates proteolytic cleavage of the receptor by a disintegrin and metalloprotease (ADAM). A subsequent cleavage by *γ*-secretase complex releases the NOTCH intracellular domain (NICD) of the receptor. NOTCH-NICD migrates into the nucleus where it binds to the recombining binding protein suppressor of hairless (RBPJ) and Mastermind-like (MAML) co-activators to assemble an active transcription complex on NOTCH-responsive genes (Figure 2).

Genes regulated by the NOTCH signalling pathway include the hairy-enhancer of split (Hes1, Hey1, Hey2) encoding the double-helical transcription factors with negative regulatory function, as well as c-Myc and cyclin D involved in cell cycle regulation [76].

The main roles of NOTCH have been associated with the regulation of homeostasis and cell proliferation, as well as the development and cell differentiation in a variety of tissues. This regulation can occur during both embryonic stages and postnatal life.

The plethora of ligands regulating NOTCH receptors have been extensively studied in different tumour types because of their onco-regulatory effects [77,78,79]. Depending on the biological microenvironment, the activation of NOTCH signalling seems to have a dual role, showing an oncogenic activity in certain tissues (i.e., the non-neuroendocrine component of small cell lung cancer) [73,80], and tumour-suppressor function in others (i.e., medullary thyroid carcinoma, small cell lung cancer, pancreatic and biliary neuroendocrine tumours) [81,82,83,84].

## 5. The Role of NOTCH Signalling in NENs

Pre-clinical studies showed a heterogeneous expression of the NOTCH receptor family in tumoral tissues, and genome sequencing analysis has identified several NOTCH gene mutations in various solid and hematological malignancies [82,85,86,87].

The main role attributed to the NOTCH signalling pathway is as a mediator of cell differentiation. Depending on the NOTCH receptor expression levels, the cross-talk with other signalling pathways and the cellular context, NOTCH signalling can have an oncogenic or tumour suppressor role [85]. In addition, alterations of the NOTCH signalling pathway are responsible for the smooth transition from a non-neuroendocrine to a neuroendocrine phenotype, as a result of a coordinated anti-cancer drug response in pathological cell conditions.

Therefore, to understand completely the impact that NOTCH operates in the development of neuroendocrine tumours, the analysis of NOTCH signalling at different layers of genomic regulation is required, ranging from gene expression levels to epigenetic alterations, and involving its diverse components as NOTCH receptors and ligands.

In the biggest cancer killers, the study of the NOTCH pathway was a milestone, and several analyses elucidated its role in pathogenesis. The expression of the different isoforms was exanimated and the presence of mutations was assessed.

In breast cancer tissue, aberrant high levels of NOTCH1 and NOTCH2 were found in comparison with control tissue [88]. Moreover, alterations in Notch signalling were also linked to triple-negative breast cancer (TNBC). Mutations were found in NOTCH1–3 at the C-terminal PEST domain, and also in the prolyl-isomerase PIN1 (peptidylprolyl cis/trans isomarase, NIMA-interacting 1) [89], supporting the theory of the involvement of Notch in breast cancer.

In some neoplasms, mutation can contribute to enhance the physiological function of the pathway, as was described in a previous non-small cell lung carcinoma (NSCLC) analysis. In this study, it was demonstrated that the presence of a C-terminal mutation in the NOTCH-1 gene confers a gain of function, increasing the receptor signalling transduction in NSCLC cancer [90].

In colorectal cancer (CRC), the genomic alteration in the NOTCH pathway correlates with clinical outcome—it may lead cells to proliferate without differentiation or to maintain the transcriptional program of normal adult colon cells. A common upregulation of the NOTCH-1 gene expression was found in tumoral samples belonging to the three different CRC transcriptional subtypes, characterized by specific transcriptional programs related to the normal adult colon, early colon embryonic development and epithelial mesenchymal transition. This finding is consistent with the critical role of Notch pathway in CRC initiation [91].

Interestingly, a recent study showed that mutation of NOTCH1 in oral squamous cell carcinoma occurs in 15% of the Caucasian population, whereas in the Asian population the rate of NOTCH1 mutations was about 50% [92]. This finding emphasizes the need to clarify the NOTCH alteration prevalence in human cancer, even more in rare neoplasms.

In NENs, the NOTCH mutational status assessment has been analysed in only a few studies, conducing whole-genome sequencing in specific neuroendocrine neoplasms.

An up-to-date one next generation sequence study was performed on the small cell neuroendocrine carcinoma of uterine cervix (SCNEC). Deyin Xing et al., found oncogenic driver mutations in KRAS, Erb-B2, c-Myc, BCL6 and NOTCH1 in a cohort of 10 small-cell neuroendocrine carcinomas (SCNEC) of the uterine cervix, a rare but extremely aggressive tumour [93]. In addition, in a cohort of large cells, neuroendocrine carcinoma (LCNEC), the most relevant molecular alteration, was detected in DLL3, a well-known NOTCH canonical ligand. The DLL3 inhibition, in combination with the use of immunotherapy, has also been pointed out as a therapeutic option for LCNEC [94]. A separate study conducted whole-genome sequencing of small cell lung cancer (SCLC), identifying inactivating mutations in the NOTCH family genes in 25% of cases [58,95].

In the following paragraphs, we summarize the current knowledge on the epigenetic modifications and NOTCH signalling pathway alteration in different types of NENs.

### 5.1. NOTCH in NENs: The Epigenetic Implications

It is conceivable to think of epigenetic changes contributing to the pathological development of tissue and how these alterations could affect gene expression after stem cell differentiation, as happens in other neoplasms. The epigenetic modifications by definition encompass all the mechanisms that modify the genetic expression and alter the genome stability, without modifying the DNA sequence. These alterations not only can occur at the chromatin level and involve acetylation and deacetylation of the histones and the methylation of the cytosine at DNA level, but can also be caused by other molecules, such as non-coding RNAs, for instance, and long non-coding RNAs and microRNAs.

Experimental data suggests that epigenetic alterations are involved in neuroendocrine tumorigenesis [96,97]. Some pivotal preclinical studies were conducted to explore the role of epigenetic alterations in NETs, obtaining interesting results—silencing regulatory genes (Wnt signaling components) and aberrant mutations in core pathways contributes in NET pathogenesis [97]. Furthermore, missense mutation in the mixed-lineage leukemia protein 3 (MLL3) often triggers aggressive neuroendocrine tumours, medulloblastomas and Merkel cell carcinoma [98] by means of inducing genomic instability.

Moreover, lysine-specific histone demethylase 1A (LSD1) inhibitor ORY-1001 was described in small cell lung cancer (SCLC) because of its anti-tumorigenic role. This inhibitor activates the NOTCH pathway, inhibiting, consequently, the transcription factor achaete–scute complex-like 1 (ASCL1), with this ultimately leading to tumorigenesis repression and to the reversion of the neuroendocrine phenotype in this type of tumour. A complete and long-term tumour regression was obtained after treating with ORY-1001 SCLC patient-derived xenograft (PDX) mice models [99]. Thus, this inhibitor has been suggested as a potential new targeted therapy for SCLC.

Recent findings on the transcriptional activation of NOTCH appear to be regulated by means of microRNAs (miRNAs), small single-stranded RNAs that regulate gene expression post-transcriptionally. Preliminary research about how aberrant miRNA expression can influence neuroendocrine cell behaviours showed a direct post-transcriptional repression of NOTCH2 and RBPJ proteins operated by miR-375 (microRNA 375) in Merkel cell carcinoma (MCC), a rare cutaneous neuroendocrine malignancy [100]. This small molecule is having an increasing connotation in the modern pathology of NEN. Arvidsson et al. discovered that miR-375 is highly expressed in small intestinal neuroendocrine tumours and could be used as prognostic biomarker for survival [101].

In the age of precision medicine, the identification of epigenetic biomarkers in a subpopulation of patients could help clinicians to choose the most appropriate therapeutic strategy. Recently, epigenetic drugs are providing promising results in preclinical phases, making attractive the idea of their use in combination with standard chemotherapy or immunotherapy. However, further validation in clinical trials is needed, and side effects have to be assessed for the possible use of these combined strategies [102].

Currently, only a few studies have focused on the epigenetic landscape in NET, and even less if we point out the implications that may occur between these epigenetic factors and the NOTCH pathway. A coordinated effort between multidisciplinary groups of experts is needed to clarify the role of NOTCH in diverse neuroendocrine neoplasms.

In the following section, we summarize the evidence gathered to date on the role of the NOTCH signalling pathway in different NENs.

### 5.2. Role of NOTCH in Neuroendocrine Tumour of the Lung

In lung tissue, the role of NOTCH has been established as driving the differentiation of neuroendocrine cells present in the organ. NOTCH mutation can provoke a dysfunction of its activity and induce neuroendocrine differentiation from no-neuroendocrine progenitors. Clinical data indicate that some neuroendocrine neoplasms of the lung could relapse and present a secondary tumour formation after anticancer therapy.

Recent findings suggest that the presence of inactivating mutations in NOTCH signalling is involved in the pathogenesis of neuroendocrine neoplasms of the lung, being defined as more than 25% of the cases for small cell lung carcinomas (SCLC) [58,103]. This fact suggests that NOTCH signalling needs to be inactivated for the development of SCLC. Moreover, NOTCH signalling is involved in the modulation of the neural and neuroendocrine differentiation process that could mean the implication of mutations in NOTCH in the neuroendocrine features of these tumours, and also in disease progression and relapse.

NOTCH pathway deregulation has been also pointed out to have a role in chemoresistance in SCLC. The effect of NOTCH in tumorigenesis seems to be done throughout the activation of the delta-like protein 3 (DLL3). Its expression is directly correlated with ASCL1 transcription factor that was found expressed in 85% of SCLCs, in contrast with an absent or minimal expression in normal lung tissue. The overexpression of DLL3 in comparison with normal tissue was also found in primary patients’ biopsies, as previously described Saunders et al. [104].

One of the features that differentiate DLL3 from other Notch ligands is its location in the Golgi that makes DLL3 able to interact with Notch1 and DLL1, blocking their transport to the endosomes for elimination and preventing them from reaching the cell surface and therefore preventing NOTCH activation. DLL3 appears to act as an inhibitor of the Notch receptor pathway.

In the mixed forms of small cell carcinomas, the modulation of the NOTCH system demonstrated the importance of this pathway in tumorigenesis and response to treatment—the activation of NOTCH reduces the particularly aggressive neuroendocrine subtype by increasing the epithelial component with a slower cell proliferation rate whose growth can be controlled with chemotherapy [73].

In summary, NOTCH acquires a tumour suppressive role through the alteration of the canonical signalling pathway in neuroendrocrine lung cancers. It could be interesting to explore the possible therapeutic strategies restoring the expression of NOTCH-mutated components in SCLC, or targeting the DDL3 in order to direct cytotoxic drugs.

### 5.3. Role of NOTCH in Neuroendocrine Gastro-Entero-Pancreatic Neuroendocrine Neoplasms (GEP-NENs)

The gastrointestinal (GI) tract and the pancreas are two of the most common sites of origin for NENs. Tumours arising from these organs are named gastro-entero-pancreatic NENs (GEP-NENs) and they represent almost the 65% of all NENs. Previously GEP-NENs were considered as a unique group of tumours, however, currently, many studies have highlighted the biological and molecular differences between pancreatic and GI NENs, as well as between the GI NENs originating from different organs of the GI tract [11,15,16,18]. Wang et al. have confirmed this heterogeneity in their study related to the implication of the NOTCH signalling pathway [105]. They demonstrated a uniform immune-histochemical expression of NOTCH1 and HES1 in well-differentiated rectal NENs—found to be, respectively, 100% and 64%—whereas only 34% and 10% of well-differentiated pancreatic NENs were positive for NOTCH1 and HES1 at immunohistochemistry, and all ileal NENs were negative to both, suggesting a possible different role of NOTCH1 in the pathogenesis of these cancers. [105].

The majority of the available studies have evaluated the role of NOTCH signalling in pancreatic NENs, thus there is a lack of knowledge on the role of NOTCH signalling in the other GEP-NENs.

In the pancreas, endocrine and exocrine cells move from a common pool of multipotent progenitors into a differentiated state, co-ordinately regulated by different mechanisms, forming together a complete and functional adult organ. After the initial developmental phase, the epithelium starts to spread pancreatic progenitor cells into different compartments: acinar cells migrated into the tips, and ductal and endocrine cells into the trunk. Endocrine cells leave the adjacent epithelia by delamination, assembling into islets of Langerhans. During the differentiation process, the mechanism of differentiation is not synchronous, and it is controlled by several regulatory agents, such as the NOTCH receptor that has an important role in the early developmental embryologic phase, as well as in adult plasticity.

In aggressive tumours of the pancreas-like pancreatic ductal adenocarcinomas (PDAC), the tumour is believed to derive from a pancreatic intraperitoneal neoplasia (PanIN). In these cases, Notch plays a dual role in the tumour initiation and development—NOTCH works as a tumour suppressor in PanIN lesions [106] and later on it has an oncogenic role in PDAC [107]. Furthermore, these studies indicate not only this dual role of the NOTCH pathway in tumorigenesis, but also the implication of several pathway components, revealing a complex fine-tuning regulation of the NOTCH pathway.

Moreover, histo-pathological studies have shown that NOTCH1 is absent or poorly-expressed in well-differentiated pancreatic neuroendocrine tumours (pNET) [8,105]. However, in MiNEN (mixed neuroendocrine/non neuroendocrine neoplasm), the expression of NOTCH1 and Hes1 is reduced or absent in the neuroendocrine cells, but both NOTCH1 and Hes1 are present in the adenomatous component [84], potentially indicating a possible role of NOTCH as a tumour suppressor gene. Further studies are needed to characterise the molecular mechanisms implicated in neuroendocrine tumorigenesis and for the understanding of the functional differences observed within pancreatic tumours.

In ileal NENs, the low or absent expression of NOTCH and HES1 has led to hypnotize a possible tumour suppressor role of the NOTCH canonical signalling cascade [105]. As confirmation of this hypothesis, Maggi et al. [108] demonstrated that Retinoblastoma-binding protein 2 (RBP2), a key component of the NOTCH repressor complex, is upregulated in gastrointestinal NENs and in liver metastases. Nonetheless, further studies are needed to confirm the role of NOTCH signalling in GI-NENs and to drive an effective therapeutic strategy modulating the NOTCH pathway in these tumours.

### 5.4. Role of NOTCH in Medullary Thyroid Cancer

Medullary thyroid cancer (MTC) is a neuroendocrine tumour that emerges from parafollicular C-cells of the thyroid gland. In MTC, the proliferation of neuroendocrine cells and tumour growth process appears to be regulated by a common pathway. A major role is played by the ASCL1 transcription factor, highly expressed in MTC, that is involved in supporting cell proliferation and embryologic precursor survival, as well as inhibiting apoptosis [109].

The canonical NOTCH signalling cascade directly reduces the expression of ASCL1, with an anti-proliferative effect. A decrease in NOTCH1 and NOTCH3 expression has been documented in MTC [81,110]. The activation of doxycycline-inducible NOTCH1 and NOTCH3 in TT cells, through treatment with increased doses of doxycycline, has demonstrated a dose-dependent increase in NOTCH1, NOTCH3 and HES1 protein, a decrease in ASCL1 levels and ultimately a reduction in tumour growth in vitro and in vivo. A decrease in the production of chromogranin A neuropeptides and specific neuron enolase (NSE), two of the main MTC biomarkers, has been also documented [81,110].

The same results were obtained by the pharmacological NOTCH3 induction, with the activating compounds AB3 suggesting NOTCH3 as a potential target for MTC treatment [111].

Moreover, the dedifferentiation process, typical of thyroid oncogenesis, seems to be correlated with the loss of NOTCH3 expression, whereas the doxycycline-induced NOTCH3 activation restores the differentiated phenotype and has an antiproliferative effect in thyroid cancer cell lines (TT, FTC) [81,111].

### 5.5. Role of NOTCH in Malignant Castration-Resistant Prostatic Cells

Prostatic small-cell carcinoma, originating from neuroendocrine diffuse cells in the prostate, is a rare neoplasia with a lack of understanding in tumour development and progression, as well as in useful prognostic factors and genetic biomarkers. More often, in prostatic cancer (PCa), prostatic cells lose the maintenance of tissue identity and by a lineage-plasticity manner transdifferentiate in the neuroendocrine phenotype following androgen deprivation therapy.

The neuroendocrine cells promote hormone-resistance, secreting peptides that can stimulate androgen-dependent growth, and reducing apoptosis. Neuroendocrine cells do not express androgenic receptors, as they are not sensitive to the therapy of androgenic deprivation and have poor sensitivity to standard chemotherapeutic agents. Interestingly, this fact seems to be related with tumour plasticity for the epithelial mesenchymal transition (EMT) process and with the alteration of the signalling pathway regulators involved in cellular proliferation and differentiation.

Currently, there are ongoing studies aimed to explore the role of NOTCH in malignant castration-resistant prostatic cancer models. Preliminary investigations revealed that hypoxia, which is linked to PCa progression, induces neuroendocrine differentiation (NED) in androgen-sensitive prostate cancer cells (LNCaP) through the downregulation of NOTCH1 and NOTCH2 mRNA and protein expression with the subsequent reduction of HES1 transcription [112]. In addition, gene profiling of castration-resistant neuroendocrine prostate cancer (NEPC) samples showed that some Notch-related genes (DLL3, DLL4, HES6, DTX1 and JAG2) are up-regulated, and others (NOTCH2 and 4) are considerably down-regulated. This fact suggests a dual role of NOTCH signalling in NEPC. Of note, in the same study, DLL3 protein expression was evaluated and it was found to be present in 76.6% of castration-resistant NEPC, but only in the 12.5% of castration-resistant prostate adenocarcinomas. This fact proposes DLL3 as a possible biomarker of NED and a potential therapeutic target for the treatment of DLL3-positive metastatic prostate cancer [113].

## 6. Therapeutic Approach Targeting NOTCH in NENs

A pharmacological modulation of the NOTCH pathway is an interesting concept to pursue for neuroendocrine tumour treatment. Overall, there are several approaches to modulate NOTCH signalling that are in different stages of development in cancer treatment. Among them, there are NOTCH-inhibiting and NOTCH-activating strategies.

The evidence gathered to date suggests that NOTCH is a tumour suppressor in NENs; however, a putative pro-oncogenic role of NOTCH signalling has been suggested for the non-neuroendocrine components of NENs and has been linked with NEN heterogeneity [73]. Thus, preliminary studies have evaluated the efficacy of both NOTCH-activating and NOTCH-inhibiting strategies.

In particular, with regard to the NOTCH-activating compounds, histone deacetylase inhibitors and valproic acid (VA) showed an in vitro antineoplastic effect in neuroendocrine cell lines (gastrointestinal carcinoid, broncopulmonary carcinoid and in human medullary thyroid cancer cell lines), inducing NOTCH1 mRNA expression through activator protein (AP) transcription factor binding [114]. In addition, in neuroendocrine cells, VA was shown to stimulate the expression of somatostatin receptor type 2 (SSTR2) that is largely targeted in neuroendocrine anticancer therapy [115]. This suggests a potential role of VA in these tumours to get a sensitization to SSTR2-targeted therapy. The efficacy of VA has also been tested in a phase II clinical trial, in which patients with G1/G2 neuroendocrine tumours treated with VA achieved relative good tumour control [116]. However, it remains unclear if the efficacy of VA in NENs is specifically related to the activation of NOTCH signalling, or if it is also related to the direct or indirect regulation of other pathways.

Therefore, a more specific targeted therapy for NOTCH signalling activation is needed. To date, there are few compounds that specifically activate NOTCH signalling and are in a very early stage of development. One example is a NOTCH3-specific antibody that binds to the NRR, causing conformational changes in the NOTCH receptor that renders S2 cleavage sites accessible to ADAMs. This antibody has been preliminary tested in 293T cells as “proof of principle” of NOTCH3 activation [117]. Thus, further studies are warranted.

The lack of big data on NOTCH-activating compounds is mainly due to the fact that in the majority of cancers, NOTCH acts as an oncogene, and, consequently, the efforts of the scientific community have been focused on the development of NOTCH-inhibiting strategies. These inhibiting strategies have been tested also in NEN treatment, studied alone or in combination with chemotherapy. The rationale behind the use of NOTCH-inhibiting drugs in NEN is mainly based on the possibility of keeping under control the non-neuroendocrine component of the tumour, preventing the neuroendocrine-to-non-neuroendocrine cell fate switch. The addition of chemotherapy is aimed to kill the neuroendocrine cells that are chemosensitive. In particular, tarextumab, a Notch2/Notch3 antagonist, has been tested alone and in combination with chemotherapy in vivo SCLC models [77], and in patients with SCLC [118]. Although in the pre-clinical models (SCLC allografts and patient-derived xenograft) the combination of tarextumab with carboplatin and irinotecan achieved a better tumour inhibition than chemotherapy or tarextumab alone [77], the clinical trial was unsuccessful, not meeting the primary endpoint of progression free survival (PFS) [118]. In an explorative clinical phase I trial, the *γ*-secretase NOTCH inhibitor RO4929097 was tested in solid malignancies, and within this trial a patient affected by colorectal cancer with neuroendocrine feature achieved a partial response [119].

Therefore, the results obtained with the NOTCH-inhibiting treatment in NENs are discordant. Future research should be aimed to select the patients, in relation to the characteristics of the tumour and the optimal timing within the course of the disease, in which the administration of NOTCH inhibitors could be most beneficial to reduce heterogeneity.

Lastly, a different approach that is neither activating nor inhibiting of NOTCH pathway, but instead targets the DLL3 NOTCH ligand, is the one behind the treatment with Rova-T. Rova-T is an antibody-drug conjugate recently tested in SCLC and neuroendocrine prostate cancer (NEPC). It is composed of a humanized monoclonal antibody directed against DLL3 and a cytotoxic payload (tesirine), and uses the DLL3 to direct the cytotoxic drug into the tumour cells. Rova-T has been tested in the phase II TRINITY trial and in the phase III TAHOE trial as third- and second-line, respectively, in patients with DLL3-positive SCLC [120,121], following the promising results of pre-clinical studies and a phase I trial [122]. In the TRINITY trial, the best overall response rate achieved with Rova-T treatment was 29% (95% confidence interval (CI), 22–36) and 16% (95% CI, 11–22) according to the investigator and the independent review committee assessment, respectively. The median overall survival was 5.6 months (95% CI, 4.9–6.8) in the overall enrolled population, while it was 6.7 in the DLL3-high (≥75% tumour cells positive for DLL3 by immunohistochemistry) patients.

Unfortunately, the enrolment in the TAHOE trial was stopped because of the shorter overall survival (OS) reported in the Rova-T arm compared with the control arm (topotecan treatment). However, despite these unfavourable results, Rova-T is currently under investigation in the phase III MERU trial (NCT03033511), which compares Rova-T to placebo as maintenance therapy after platinum-based chemotherapy in patients with extensive-stage SCLC. In addition, phase I studies are also evaluating Rova-T in combination with first-line chemotherapy (cisplatin/etoposide), nivolumab and nivolumab plus ipilimumab in NSLC. Rova-T is also under investigation as treatment for patients with NEPC (NCT02709889), following the excellent pre-clinical results [123]. The results of these trials are strongly awaited.

The evidence gathered to date is not strong enough to draw a general conclusion on the optimal NOTCH modulatory strategy to be pursued for NEN treatment. However, it seems that NOTCH inhibition could be needed to reduce tumour heterogeneity within the NENs; however, once the neuroendocrine phenotype is established, NOTCH activation should be targeted. A limitation of the NOTCH-activating approach is the possible off-target oncogenic effect on other tissues that could result in higher damage to the patient. Thus, strategies to ensure a high target specificity are required. Two possible attractive solutions to this problem could be the development of bi-specific antibodies and/or functionalized nanoparticles carrying a NOTCH activator [124,125]. In the first approach, antibodies are generated to bind to two different antigens, with one usually being the therapeutic target and the other being a specific marker of the tumour to treat. This conformation ensures specific therapeutic activity at the tumour side, ultimately reducing the off-target effect. For the NEN treatment, a possible bi-specific antibody could target epidermal growth factor receptor (EGFR) with an antagonistic effect and NOTCH with an activating effect. A similar approach has already been successfully tested in other cancers, but instead using an EGFR/NOTCH antagonist antibody [126]. In the second approach, nanoparticles are functionalized with different peptides to efficiently deliver the targeted drug that is loaded into the nanoparticle itself. An attractive hypothesis for the treatment of somatostatin receptor positive NEN treatment would be functionalize the nanoparticle-carrying NOTCH activator with tumour-inhibiting somatostatin analogues. However, this represents a preliminary hypothesis that must first be tested.

## 7. Conclusions

Although several studies have been conducted with the aim of identifying genetic mutations involved in the genesis of neuroendocrine tumours, none of them have shown a substantial mutational percentage in the samples analysed, revealing a low-abundance of consistent mutations in G1/G2 neoplasms compared with other malignancies. Moreover, the modern sequencing technologies highlighted heterogeneity of mutations depending on the tumour anatomic origin.

For these reasons, there are still many unknowns in the genomic characterization of NETs in comparison with other neoplasia, with the majority of the data covering predominantly pancreatic, lung and small-intestine NET.

Therefore, multi-centre collaborations, international databases, biological banks and genome-wide profiling overture should be pursued.

The NOTCH pathway has recently emerged as key factor in NEN development and progression, and it seems to play an important role in generating NEN intra-tumour heterogeneity. NOTCH signalling mainly has a tumour-suppressive role in NENs, justifying the use of NOTCH-activating strategies for their treatment. However, a proto-oncogenic role has been suggested in the non-neuroendocrine components of NENs that generates a fertile microenvironment for the development of neuroendocrine tumours, thus also the NOTCH-inhibiting approach has been considered in NEN treatment.

To date, both NOTCH-activating and NOTCH-inhibiting approaches are still at a very early phase of development as therapy for NENs, whereas more advanced but debatable results have been achieved with Rova-T treatment.

In conclusion, the role of NOTCH as a therapeutic target in NENs, as well as the currently available therapeutic strategies for targeting this pathway, have to be further investigated and developed. In particular, future research should be aimed at elucidating the possible specific targets within the NOTCH pathway susceptible to pharmacological regulation, and to identify specific tumour markers that could be used to deliver NOTCH-modulator agents to the tumour cells, as well as unveil specific biomarkers that could better stratify the group of patients that could benefit from a NOTCH-activating and/or inhibiting therapy.
